# Sepsis-Like Presentation of Tuberculous Meningitis With Rapid Neurologic Decline and Spinal Subdural Abscesses: A Case Report

**DOI:** 10.7759/cureus.102908

**Published:** 2026-02-03

**Authors:** Risit Datta, Amber Jin, Cesar R Acosta, Kaung Thu, Roxana Lazarescu

**Affiliations:** 1 Internal Medicine, Touro College of Osteopathic Medicine, New York, USA; 2 Internal Medicine, Wyckoff Heights Medical Center, New York, USA

**Keywords:** magnetic resonance imaging, rapid quadriparesis, sepsis-like illness, spinal subdural abscess, tuberculous meningitis (tbm)

## Abstract

Tuberculous meningitis (TBM) is the most severe form of central nervous system tuberculosis and carries high morbidity and mortality. We report a 35-year-old man from Ecuador who presented with sepsis-like systemic symptoms and rapidly progressive neurological deficits. Initial imaging revealed cavitary pulmonary lesions and hilar lymphadenopathy, suggestive of pulmonary tuberculosis. Neurological deterioration prompted magnetic resonance imaging (MRI) of the brain and cervical spine, demonstrating communicating hydrocephalus, basilar leptomeningeal enhancement, diffuse cervical cord edema, and multiple rim-enhancing subdural abscesses. Diagnosis was confirmed via bronchoalveolar lavage positive for *Mycobacterium tuberculosis* after multiple failed sputum induction attempts. This case illustrates a combination of sepsis-like presentation, rapid quadriparesis, and spinal cord abscesses in TBM, highlighting the importance of advanced imaging and alternative microbiologic sampling for timely diagnosis and management.

## Introduction

Tuberculous meningitis (TBM) arises when *Mycobacterium tuberculosis *disseminates from a primary pulmonary focus to the central nervous system. Subependymal collections, known as Rich foci, may rupture into the subarachnoid space, triggering a granulomatous inflammatory response. This process results in thick gelatinous exudates within the basal cisterns that can encase cranial nerves and cerebral vessels, leading to vasculitis, cerebral infarction, and obstruction of cerebrospinal fluid (CSF) flow with subsequent hydrocephalus. Both innate and adaptive immune responses contribute to blood-brain barrier disruption and neuronal injury, compounding disease severity [1,2].

Clinically, TBM classically presents with a subacute course of headache, fever, meningeal irritation, and altered mental status. However, manifestations can vary widely depending on host factors, immune status, and disease burden, and adults may lack overt meningeal signs or present with nonspecific systemic symptoms [3,4]. These atypical presentations pose significant diagnostic challenges and frequently delay the initiation of anti-tuberculous therapy, which is closely associated with worse neurological outcomes and increased mortality. In particular, TBM may rarely mimic a sepsis-like syndrome, obscuring the underlying central nervous system infection. This case highlights such an atypical adult presentation and underscores the need for heightened clinical suspicion of TBM in patients with unexplained systemic inflammatory states and progressive neurological decline.

## Case presentation

A 35-year-old man originally from Ecuador with no significant past medical history presented with several months of progressive fatigue, malaise, and unintentional weight loss of approximately 8 kg. Four days prior to presentation, he developed acute gastrointestinal symptoms, including multiple episodes of non-bloody, non-bilious vomiting, generalized periumbilical abdominal pain, dizziness, and generalized weakness. He also reported decreased oral intake, mild anorexia, and intermittent subjective fevers. He denied cough, hemoptysis, night sweats, recent travel, or known tuberculosis exposure. Neurologically, he endorsed new-onset slurred speech, hand tremors, unsteady gait, and a single episode of urinary incontinence. He denied prior neurological or urinary dysfunction. He reported no tobacco or alcohol use and only occasional marijuana use.

On arrival, the patient appeared ill but was alert, oriented, and well-nourished. Vital signs were notable for tachycardia (heart rate 123 bpm) with normotension and afebrile status. Cardiopulmonary examination was unremarkable aside from tachycardia. Abdominal examination demonstrated diffuse tenderness, most prominent in the periumbilical region, without guarding or rebound. A markedly distended bladder was noted; Foley catheter placement drained 2.1 liters of urine, consistent with acute urinary retention. Neurological examination revealed mild, diffuse weakness without clear focal deficits, along with hand tremors and an unsteady gait. There were no signs of meningeal irritation on initial assessment.

**Figure 1:**
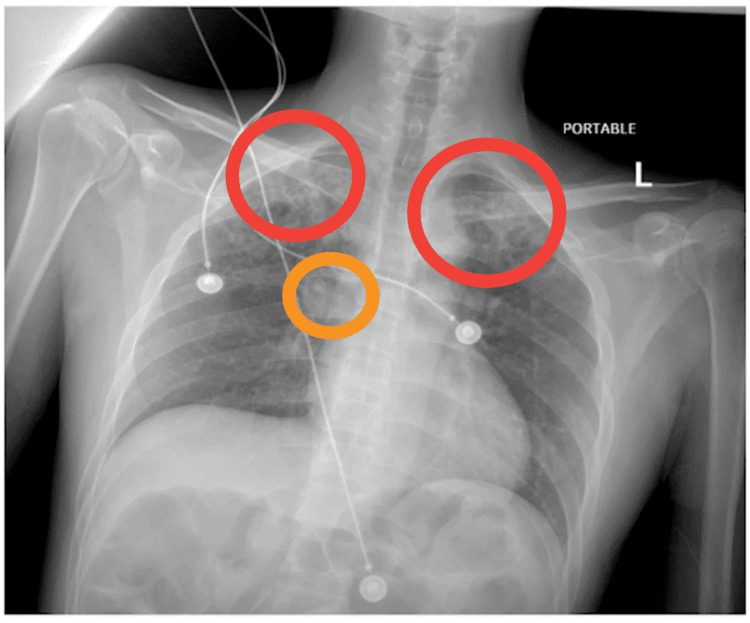
Chest radiograph demonstrating bilateral upper-lobe opacities with associated right hilar lymphadenopathy measuring approximately 2 cm Red: Bilateral upper-lobe opacities. Orange: Right hilar lymphadenopathy measuring approximately 2 cm

Initial laboratory evaluation showed leukocytosis (WBC 14,000/μL with 89.7% neutrophils) and normal renal function. Human immunodeficiency virus (HIV) testing was negative. Given the combination of systemic inflammatory findings, tachycardia, and pulmonary symptoms suggested by imaging, the initial differential diagnosis included sepsis secondary to community-acquired pneumonia, pulmonary tuberculosis, and septic emboli. Chest radiography revealed bilateral upper-lobe opacities with right hilar lymphadenopathy measuring approximately 2 cm (Figure 1).

**Figure 2:**
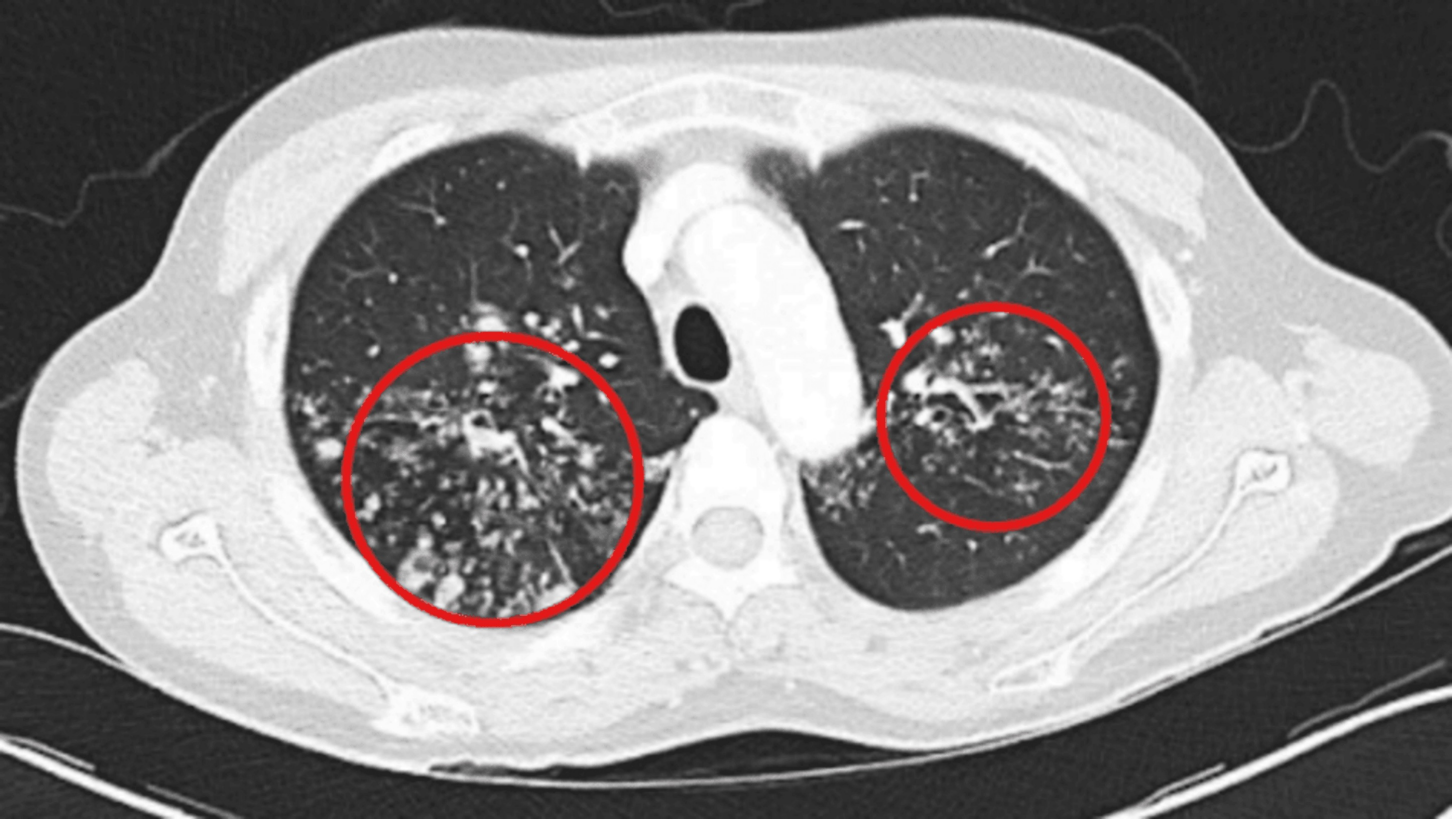
Computed tomography of the chest demonstrating innumerable solid pulmonary lesions with multiple large cavitary areas predominantly involving the upper lobes

Subsequent computed tomography (CT) of the chest demonstrated innumerable solid pulmonary nodules with multiple large cavitary lesions predominantly involving the upper lobes (Figure 2).

These findings raised strong concern for post-primary pulmonary tuberculosis, though alternative considerations such as necrotizing bacterial pneumonia and septic emboli were also considered.

**Figure 3:**
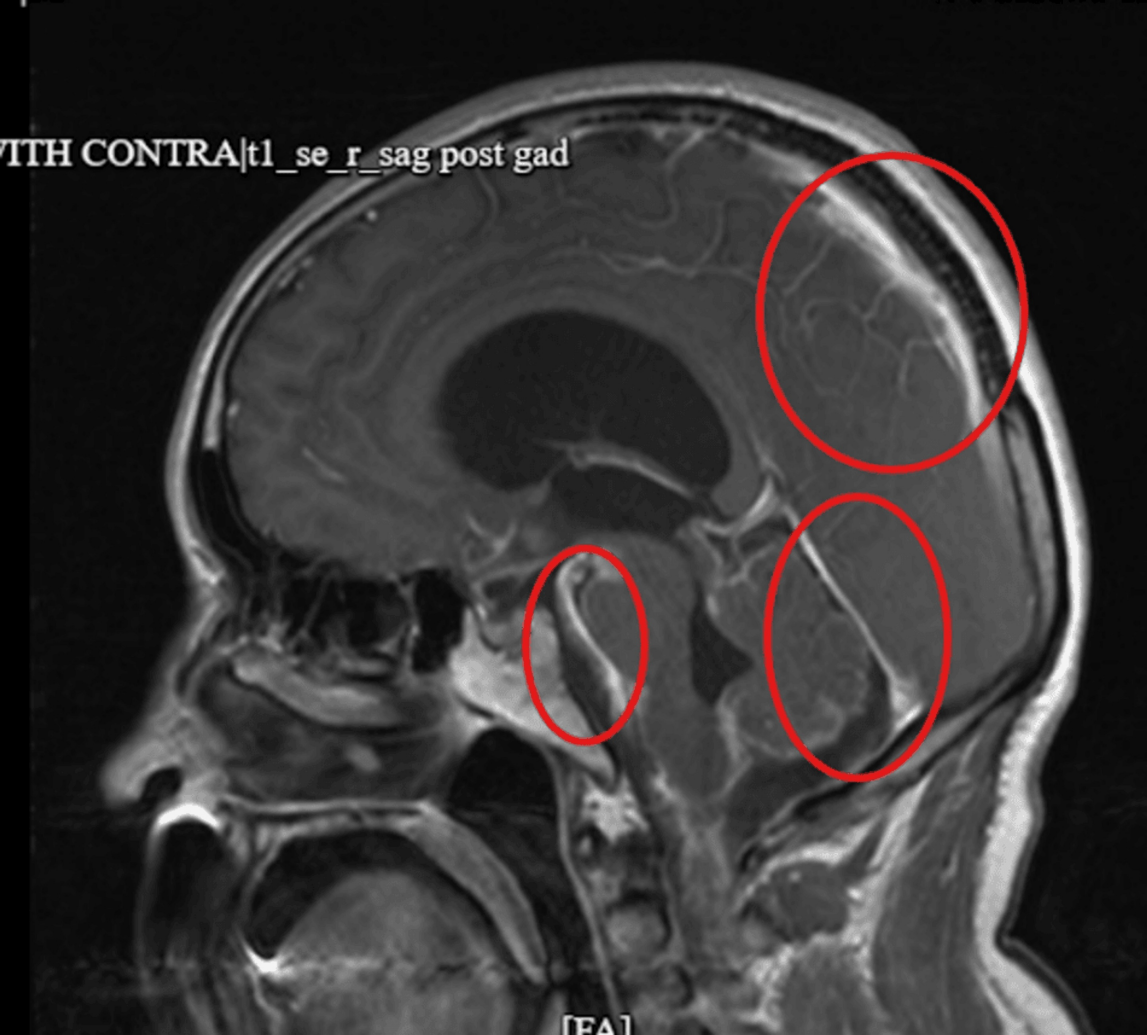
Brain magnetic resonance imaging demonstrating communicating hydrocephalus with transependymal cerebrospinal fluid flow and multiple enhancing lesions surrounding the brainstem and basilar cisterns with diffuse subarachnoid enhancement consistent with a leptomeningeal process

Despite multiple sputum induction attempts, acid-fast bacilli (AFB) testing was initially unsuccessful. During hospitalization, the patient developed progressive neurological deficits, including worsening gait instability and persistent urinary retention, prompting further evaluation for central nervous system pathology. Magnetic resonance imaging (MRI) of the brain revealed communicating hydrocephalus with transependymal CSF flow, suggestive of impaired CSF resorption, and multiple enhancing lesions surrounding the brainstem and basilar cisterns with diffuse leptomeningeal enhancement consistent with an inflammatory meningeal process (Figure 3).

**Figure 4:**
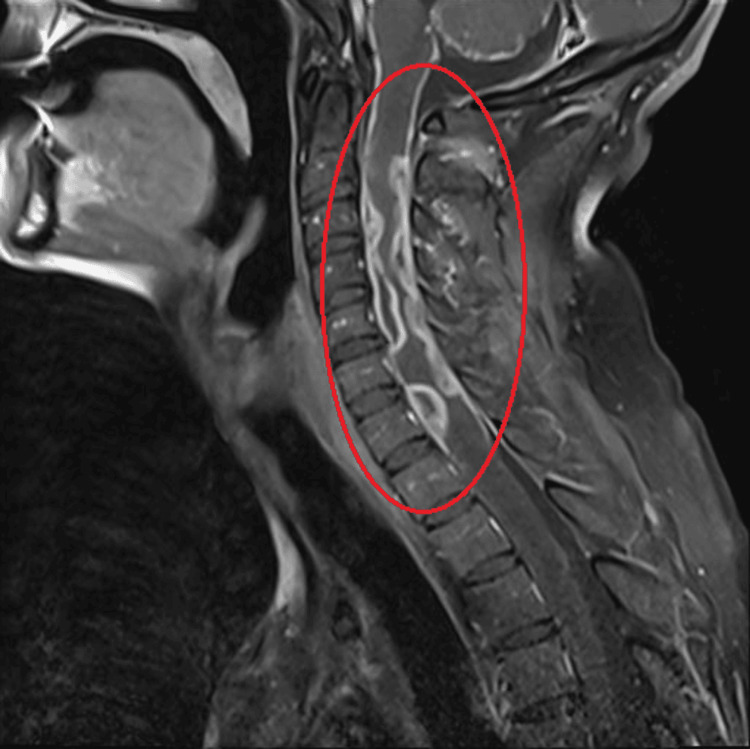
Magnetic resonance imaging of the cervical spine demonstrating diffuse cord edema and multiple rim-enhancing subdural abscesses

MRI of the cervical spine demonstrated diffuse spinal cord edema, multiple rim-enhancing subdural collections consistent with subdural abscesses, and subtle paravertebral inflammatory changes, findings concerning for spinal involvement of disseminated infection (Figure 4).

Lumbar puncture was performed following neuroimaging and demonstrated mild lymphocytic pleocytosis. However, CSF AFB staining and cultures were negative. Although these findings were nondiagnostic, TBM remained high on the differential given the characteristic neuroimaging findings, subacute clinical course, and evidence of disseminated disease. Bronchoscopy with bronchoalveolar lavage was subsequently performed and returned positive for *Mycobacterium tuberculosis*, confirming the diagnosis of disseminated tuberculosis with central nervous system involvement.

The patient was initiated on standard four-drug anti-tuberculous therapy with isoniazid, rifampin, pyrazinamide, and ethambutol, along with adjunctive intravenous dexamethasone due to central nervous system involvement. Supportive management included bladder catheterization, early physical therapy, and close monitoring for medication-related adverse effects. Neurosurgical consultation was obtained for hydrocephalus. However, external ventricular drainage or shunt placement was deferred given stable intracranial pressures and close clinical monitoring. Over the first two weeks of treatment, the patient demonstrated gradual improvement in upper extremity strength and partial recovery of urinary function, though residual lower extremity weakness persisted.

## Discussion

This case highlights several uncommon and educational features of TBM that expand its recognized clinical spectrum and underscore important diagnostic and management considerations. While atypical presentations of TBM have been described, this case is notable for the convergence of multiple rare features occurring simultaneously, resulting in diagnostic complexity and rapid neurological deterioration.

First, the patient's initial presentation closely resembled systemic sepsis, characterized by tachycardia, leukocytosis, malaise, and gastrointestinal symptoms rather than classic meningeal signs. Adult TBM most commonly presents with headache, fever, neck stiffness, and altered mental status, typically evolving over weeks rather than days. Sepsis-like presentations are infrequently reported in immunocompetent adults and may delay the diagnostic consideration of TBM, particularly in the absence of respiratory symptoms or overt meningismus [5]. In this case, the absence of headache, photophobia, or neck stiffness likely contributed to early diagnostic uncertainty and broadened the initial differential to include community-acquired pneumonia and septic emboli. This underscores the importance of maintaining suspicion for TBM in patients from endemic regions who present with unexplained systemic inflammation and weight loss, even in the absence of classic neurologic features.

Second, the patient experienced unusually rapid neurological progression, advancing within days from mild gait instability to quadriparesis and neurogenic bladder. TBM typically follows a subacute course, with gradual development of cranial neuropathies, cognitive impairment, or focal deficits over several weeks. Rapid neurological decline, as observed here, suggests aggressive central nervous system dissemination with extensive leptomeningeal inflammation and early spinal cord involvement [3,4,6]. This rapid progression has important prognostic implications, as early neurologic deterioration in TBM is strongly associated with poorer functional outcomes despite appropriate therapy.

Third, this case is distinguished by extensive spinal involvement, including rim-enhancing spinal subdural abscesses. While basal meningeal exudates, hydrocephalus, and parenchymal tuberculomas are well-recognized features of TBM, spinal subdural abscesses remain rare and are more often reported in immunocompromised hosts or in association with advanced disease [7-9]. In this patient, concurrent cranial and spinal pathology likely accounted for the rapid development of quadriparesis and urinary retention. These findings emphasize the diagnostic value of imaging the entire neuroaxis in TBM patients who exhibit spinal cord symptoms, bladder dysfunction, or rapidly progressive weakness, as isolated brain imaging may underestimate disease burden.

Fourth, the diagnostic process was complicated by repeatedly negative sputum studies and inconclusive CSF analysis. CSF findings in TBM are often nonspecific and may include only mild lymphocytic pleocytosis early in the disease course, with low sensitivity for AFB staining and culture [1,2]. In this case, microbiologic confirmation required bronchoalveolar lavage, highlighting the importance of pursuing alternative sampling strategies when clinical suspicion remains high. This reinforces a key diagnostic principle in TBM: confirmation often depends on integrating epidemiologic risk factors, characteristic imaging findings, and microbiologic data from multiple sources rather than reliance on CSF studies alone.

From a management perspective, this case reinforces the critical role of early empiric anti-tuberculous therapy and adjunctive corticosteroids once TBM is suspected. Dexamethasone has been shown to reduce mortality and neurologic sequelae by mitigating inflammatory-mediated cerebral edema, vasculitis, and infarction [10,11]. Advanced neuroimaging was instrumental not only in establishing the diagnosis but also in identifying complications such as hydrocephalus and spinal abscesses, which guided neurosurgical consultation and close monitoring. Although CSF diversion was ultimately deferred due to stable intracranial pressures, early neurosurgical involvement was essential given the high risk of rapid decompensation.

In summary, this case is notable for the combination of four uncommon features: (1) a sepsis-like systemic presentation without classic meningitic symptoms, (2) rapid neurologic decline to quadriparesis and neurogenic bladder, (3) simultaneous cranial and spinal central nervous system involvement including rim-enhancing subdural abscesses, and (4) the need for bronchoalveolar lavage to establish microbiologic diagnosis after negative sputum and CSF studies. Together, these features highlight the diagnostic challenges of TBM and underscore the need for early comprehensive imaging, broad diagnostic strategies, and prompt empiric treatment to reduce morbidity and prevent irreversible neurologic injury.

## Conclusions

This case underscores the diagnostic challenges of TBM presenting with sepsis-like systemic symptoms, rapid neurological deterioration, and combined cranial and spinal involvement. Clinicians should maintain suspicion for TBM in patients from endemic regions with unexplained systemic inflammation or early neurologic deficits, even in the absence of classic meningeal signs. Prompt neuroaxis imaging, use of alternative microbiologic sampling when CSF studies are nondiagnostic, and early initiation of anti-tuberculous therapy with adjunctive corticosteroids are critical to reducing morbidity and preventing irreversible neurologic injury.
